# Efficient production of vindoline from tabersonine by metabolically engineered *Saccharomyces cerevisiae*

**DOI:** 10.1038/s42003-021-02617-w

**Published:** 2021-09-16

**Authors:** Tengfei Liu, Ying Huang, Lihong Jiang, Chang Dong, Yuanwei Gou, Jiazhang Lian

**Affiliations:** 1 Key Laboratory of Biomass Chemical Engineering of Ministry of Education, College of Chemical and Biological Engineering, Zhejiang University, Hangzhou, 310027 China; 2 Hangzhou Global Scientific and Technological Innovation Center, Zhejiang University, Hangzhou, 310027 China; 3 School of Chinese Materia Medica, Beijing University of Chinese Medicine, Beijing, 100102 China

**Keywords:** Metabolic engineering, Applied microbiology

## Abstract

Vindoline is a plant derived monoterpene indole alkaloid (MIA) with potential therapeutic applications and more importantly serves as the precursor to vinblastine and vincristine. To obtain a yeast strain for high yield production of vindoline from tabersonine, multiple metabolic engineering strategies were employed via the CRISPR/Cas9 mediated multiplex genome integration technology in the present study. Through increasing and tuning the copy numbers of the pathway genes, pairing cytochrome P450 enzymes (CYPs) with appropriate cytochrome P450 reductases (CPRs), engineering the microenvironment for functional expression of CYPs, enhancing cofactor supply, and optimizing fermentation conditions, the production of vindoline was increased to a final titer as high as ∼16.5 mg/L, which is more than 3,800,000-fold higher than the parent strain and the highest tabersonine to vindoline conversion yield ever reported. This work represents a key step of the engineering efforts to establish de novo biosynthetic pathways for vindoline, vinblastine, and vincristine.

## Abstract (web summary)

Liu et al. optimize vindoline biosynthesis in yeast from tabersonine by altering the copy numbers of pathway genes, pairing CYPs with CPRs, engineering the microenvironment for functional expression of CYPs, enhancing cofactor supplies, and optimizing fermentation conditions. By doing so, the authors increased the production of vindoline to a final titer as high as ∼16.5 mg/L.

## Introduction

Vindoline is a monoterpene indole alkaloid (MIA) derived from *Catharanthus roseus*, one of the most extensively investigated medicinal plants. Vindoline serves as both the biosynthetic and synthetic precursor of the therapeutically important anticancer drugs, vinblastine, and vincristine^1,2^. However, the low yield of these MIAs in plant as well as the difficulty in chemical synthesis result in high market demands with exorbitant price^3^. To overcome these drawbacks, several studies have shown that metabolically engineered yeasts are promising cell factories for the production of plant-derived alkaloids, such as sanguinarine^4^, strictosidine^5^, noscapine^6^, tropine^7^, and scopolamine^8^. For example, a combination of enzyme engineering, pathway and strain engineering, and fermentation optimization led to the production of noscapine at a titer of ∼2.2 mg/L in shake flasks^6^. In another study, enhancing the supply of precursors, removing competing pathways, and increasing the copy numbers of rate-limiting enzyme encoding genes were employed to achieve de novo production of strictosidine, with a final titer as high as ∼0.5 mg/L^5^.

**Fig. 1:**
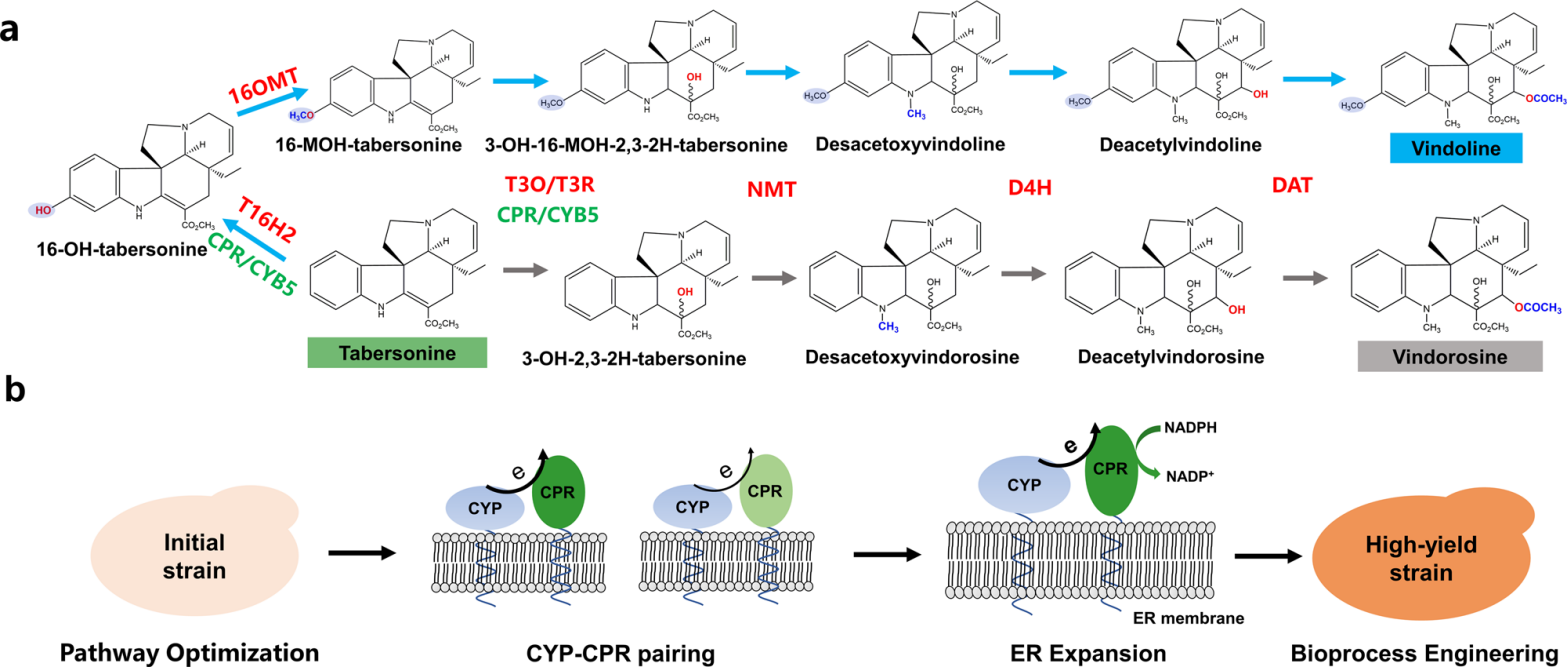
Construction of a vindoline-producing yeast strain. **a** Metabolic pathway for the biosynthesis of vindoline/vindorosine from tabersonine. Eight plant-derived enzymes should be functionally expressed in yeast to reconstitute the vindoline biosynthetic pathway. *T16H2*: tabersonine 16-hydroxylase 2; *16OMT*: 16-hydroxytabersonine O-methyltransferase; *T3O*: tabersonine 3-oxygenase; *T3R*: tabersonine 3-reductase; *NMT*: 3-hydroxy-16-methoxy-2,3-dihydrotabersonine-N-methyltransferase; *D4H*: desacetoxyvindoline-4-hydroxylase; *DAT*: deacetylvindoline-4-O-acetyltransferase; *CPR*: cytochrome P450 reductases; 16-OH-tabersonine: 16-hydroxytabersonine; 16-MOH-tabersonine: 16-methoxytabersonine; 3-OH-16-MOH-2,3-2H-tabersonine: 3-hydroxy-16-methoxy-2,3-dihydro-tabersonine; 3-OH-2,3-2H-tabersonine: 3-hydroxy-2,3-dihydro-tabersonine. **b** Brief engineering strategies for the construction of an efficient *S. cerevisiae* cell factory for high-yield production of vindoline from tabersonine. Different colors represented CPRs with different origins, e represented the electron transfer between CYPs and CPRs.

Recently, the entire 31-step biosynthetic pathway from geranyl pyrophosphate to vindoline has been fully elucidated in *C. roseus*^9–15^. The biosynthesis of vindoline from tabersonine (Fig. 1a) starts from the hydroxylation by the tabersonine 16-hydroxylase 2 (T16H2), followed by O-methylation by the 16-hydroxytabersonine O-methyltransferase (16OMT), resulting in the formation of 16-hydroxytabersonine and 16-methoxytabersonine, respectively. 16-Methoxytabersonine is then sequentially catalyzed by T3O (tabersonine 3-oxygenase; CYP71D1V2)/T3R (tabersonine 3-reductase; ALDH1), NMT (3-hydroxy-16-methoxy-2,3-dihydrotabersonine-N-methyltransferase), D4H (desacetoxyvindoline-4-hydroxylase), and DAT (deacetylvindoline-4-O-acetyltransferase), leading to the biosynthesis of vindoline. Noteworthy, as several cytochrome P450 enzymes (CYPs) were involved in vindoline biosynthesis (i.e., T16H2 and T3O), cytochrome P450 reductases (CPRs) should be included as electron transfer partners. In addition, cytochrome b5 (CYB5), serving as an electron transfer link between NADPH and cytochrome, was often co-expressed with CPRs to enhance electron transfer efficiency, and accordingly the enzymatic activity of CYPs and the production of natural products^16^. Due to enzyme promiscuity, vindorosine was synthesized by the same pathway (Fig. 1a), which is considered as the biggest challenge and should be minimized for high-yield production of vindoline^17^. Although the tabersonine to vindoline conversion pathway has been elucidated and reconstituted, the titer and conversion yield was extremely low, which hampered the reconstitution of de novo biosynthetic pathways to produce vindoline, vinblastine, and vincristine. In other words, metabolic engineering efforts should be devoted to improving the titer and yield of vindoline.

In the present study, *Saccharomyces cerevisiae* was employed as a host for the reconstitution of the biosynthetic pathway of vindoline from tabersonine. Multiple metabolic engineering efforts were made to achieve high-yield biosynthesis of vindoline. Initially, the reconstitution of the whole biosynthetic pathway (*T16H2*, *16OMT*, *T3O*, *T3R*, *NMT*, *D4H*, *DAT*, *CPR*, and *CYB5* from *C. roseus*) with one chromosomal copy for each gene resulted in marginal production of vindoline in yeast. Followed by increasing the expression levels of the rate-limiting enzyme encoding genes, pairing CYPs with appropriate CPRs, modifying the microenvironment for CYPs functional expression, enhancing cofactor supply, and optimizing fermentation conditions (Fig. 1b), the production of vindoline was increased for more than ~3,800,000-fold, with a final titer as high as ∼16.5 mg/L. The vindoline-producing yeast strain constructed in this study not only serves as a classic example of metabolic engineering for producing value-added secondary metabolites (i.e., plant natural products), but also takes a critical step in establishing de novo biosynthetic pathways to produce vindoline, vinblastine, and vincristine.

## Results

### Reconstitution of the vindoline biosynthetic pathway in yeast

**Fig. 2:**
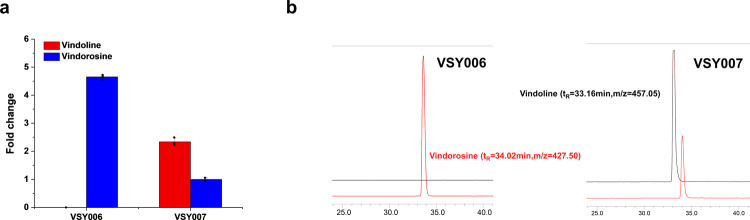
Comparison of vindoline and vindorosine production in engineered *S. cerevisiae* strains. **a** Productivity changes of vindoline and vindorosine in strain VSY006 and VSY007, with the production level of vindorosine in strain VSY006 set as the reference value. **b** MS spectra of vindoline and vindorosine produced by strain VSY006 and VSY007, respectively. Error bars represented SD of biological triplicates (*n* = 3).

As the biosynthetic pathway from tabersonine has been fully elucidated with several CYPs involved (i.e., T16H2 and T_3_O, Fig. 1a), VSY002, in which *CrCPR* and *CrCYB5* had been integrated, was firstly constructed for the introduction of vindoline biosynthetic pathway. Unfortunately, the production of vindoline in strain VSY006, containing a single copy of each gene of the whole vindoline biosynthetic pathway, was nearly undetectable with MRM mode or scan mode (as low as ~4.2 ng/L). On the contrary, the by-product vindorosine, which could be synthesized from tabersonine by the downstream pathway (T3O/T3R, NMT, D4H, and DAT), was accumulated to high levels (Fig. 2). These results indicated that the T16H2 and 16OMT activities might be the bottlenecks for vindoline biosynthesis, which was consistent with the previous study^17^. To verify the hypothesis, the plasmid pESC-LEU2d-T16H2-16OMT was transformed into the VSY006 strain. Compared with VSY006, the introduction of *T16H2* and *16OMT* on a multi-copy plasmid (strain VSY007) significantly increased the vindoline production (13.8 μg/L). Meanwhile, the accumulation of vindorosine was dramatically decreased and the production level was ~2.34 fold lower than that of vindoline (Fig. 2a). The LC-MS SIM mode profiles clearly indicated the metabolic shift from vindorosine (*t*_R_ = 34.02 min, *m*/*z* = 427.05) accumulation in VSY006 to vindoline (*t*_R_ = 33.16 min, *m*/z = 457.05) biosynthesis in VSY007 (Fig. 2b). These results revealed that increasing the copy numbers of *T16H2* and *16OMT* was beneficial for converting tabersonine to vindoline.

### Improving vindoline production via biosynthetic pathway optimization

**Fig. 3:**
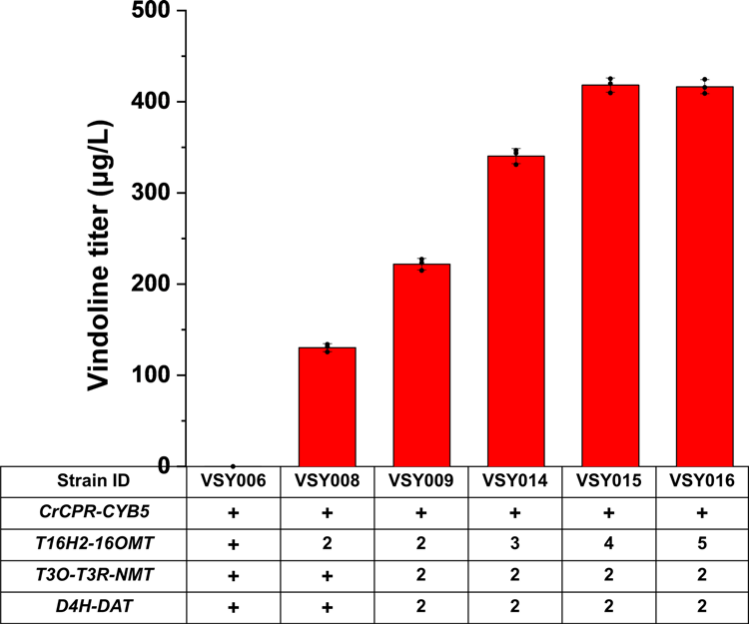
Enhancing vindoline biosynthesis via increasing and tuning the copy numbers of the rate-limiting enzyme encoding genes. With VSY006 as the parent strain, additional copies of *T16H2*-*16OMT* expression cassettes and other pathway gene expression cassettes were introduced by CRISPR/Cas9-mediated multi-copy genome integration technology. Error bars represent SD of biological triplicates (*n* = 3).

Considering that higher copy numbers of *T16H2* and *16OMT* were required for vindoline production and the plasmid system was not suitable for practical applications, multiplex and multi-copy genome integration of the biosynthetic pathway genes was employed using the CRISPR/Cas9-mediated genome editing tool (Supplementary Fig. S1). As shown in Fig. 3, the integration of another copy of *T16H2* and *16OMT* expression cassettes into strain VSY006 increased the production of vindoline to 130.3 μg/L (VSY008), which was also significantly higher than that of the plasmid system (strain VSY007), indicating the advantage of genome integration for cell factory development. Further integration of another copy of the other pathway genes into VSY008 resulted in the construction of VSY009, which harbored two copies for each gene of the vindoline biosynthesis pathway. The production of vindoline in strain VSY009 was increased ~1.71-fold to 221.9 μg/L, when compared with strain VSY008. However, vindorosine was still accumulated to high levels, indicating that T16H2 and 16OMT remained the bottlenecks for high-yield conversion of tabersonine to vindoline.

To further enhance 16-hydroxytabersonine and 16-methyoxytabersonine synthesis and accordingly direct the metabolic flow toward vindoline production, additional copies of *T16H2* and *16OMT* were further integrated into VSY009, resulting in the construction of VSY014 (three copies of *T16H2* and *16OMT*), VSY015 (four copies of *T16H2* and *16OMT*), and VSY016 (five copies of *T16H2* and *16OMT*). VSY015 harboring four copies of *T16H2* and *16OMT* and two copies of the remaining pathway genes resulted in the highest production of vindoline, which was 3.2- and 1.88-fold higher than that of VSY008 and VSY009, respectively. Integration of extra copies of *T16H2* and *16OMT* failed to further increase the conversion of tabersonine into vindoline (Fig. 3). Therefore, the biosynthetic pathway with four copies of *T16H2* and *16OMT* and two copies of *T3O*, *T3R*, *NMT*, *D4H*, and *DAT* were optimal for vindoline conversion, and VSY015 was chosen for subsequent optimization.

### Engineering CYPs microenvironment and increasing co-substrate availability for increased vindoline production

As mentioned above, two plant CYPs (T16H2 and T3O) were involved in vindoline biosynthesis. Considering the pivotal role of CPRs in NADPH-mediated electron transfer and the requirement of endoplasmic reticulum (ER) membrane localization for CYPs activities, the following strategies were employed to improve the microenvironment of CYPs for optimal performance: (1) CYP-CPR pairing; (2) ER expansion; (3) NADPH supply enhancement.

**Fig. 4:**
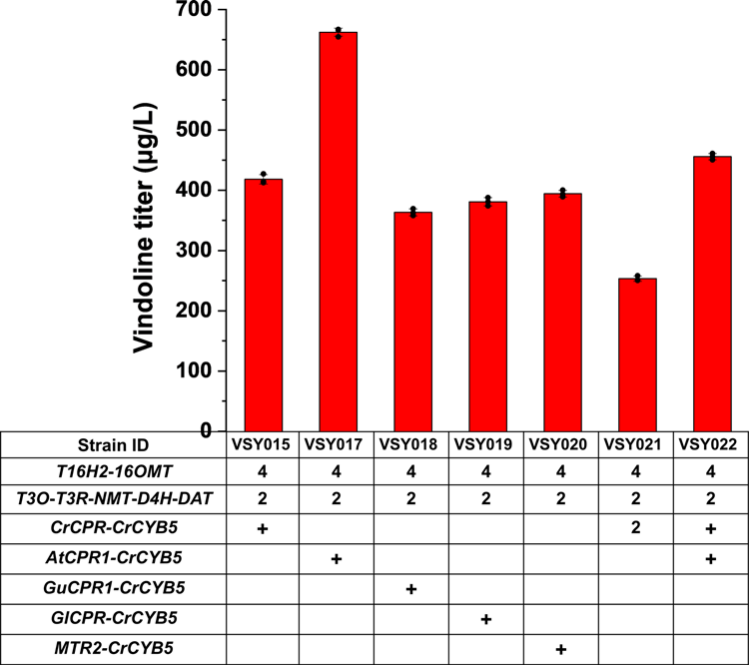
Combinatorial optimization of CYPs-CPRs pairing to improve vindoline production. Five CPRs with different origins (*CrCPR* from *C. roseus*, *AtCPR1* from *A. thaliana*, *GuCPR1* from *G. uralensis*, *GlCPR* from *G. lucidum*, and *MTR2* from *Medicago*) were integrated into the yeast genome and their effects on vindoline production were investigated. All strains were cultured in SC with 2% galactose in the presence of 50 mg/L tabersonine. Error bars represent SD of biological triplicates (*n* = 3).

Previous studies demonstrated that the source of CPRs could affect the activities of CYPs and the native CPRs were not necessarily the best partner for CYPs in a heterologous host^18–20^. Therefore, CYPs should be appropriately paired with CPRs for optimal function and accordingly vindoline biosynthesis. In yeast strain VSY015, the native CPR from *C. roseus* (*CrCPR*) was integrated. To investigate the effect of CYPs-CPRs pairing on vindoline biosynthesis, four different CPR-encoding genes including *AtCPR1* (*Arabidopsis thaliana*), *GuCPR1* (*Glycyrrhiza uralensis*), *GlCPR* (*Ganoderma lucidum*), and *MTR2* (*Medicago*) were chosen to replace *CrCPR* in VSY015, resulting in the construction of VSY017, VSY018, VSY019, and VSY020, respectively. The replacement of *CrCPR* with *AtCPR1* resulted in significant improvements in vindoline production (∼1.8-fold, 662.4 μg/L), while a comparable titer was observed for all the remaining CPR replacement (Fig. 4). Interestingly, the integration of an additional copy of *CrCPR* into VSY015 and VSY017 resulted in decreased vindoline production, 253.4 μg/L in VSY021 and 455.8 μg/L in VSY022, respectively.

As AtCPR1 demonstrated the best compatibility with the two *C. roseus* CYPs (T16H2 and T3O), an additional copy of *AtCPR1* was integrated into VSY017. Unfortunately, the production of vindoline was decreased in VSY017-2 (Supplementary Fig. S2), indicating the benefits of overexpression of *AtCPR1* was counteracted by the endogenous CPR (ScCPR), encoded by *NCP1*^21^. ScCPR has been found to possess low compatibility with plant CYPs and might interfere with the electron transfer between AtCPR1 and *C. roseus* CYPs (T16H2 and T3O). Therefore, to further explore the CYP-CPR interaction for optimal catalytic activity of CYPs, *NCP1* was deleted and the effect on vindoline production was investigated in the present study. VSY017 was further engineered by inserting *CPRs* and *CrCYB5* expression cassettes into the *NCP1* locus, resulting in the construction of VSY017-3, VSY017-4, VSY017-5, VSY017-6, and VSY017-7, respectively. As expected, the integration of an additional copy of *CPRs* into the *NCP1* locus of VSY017 increased vindoline production, with the highest production achieved in VSY017-3 (1264.2 μg/L), representing a two-fold increase over the parental strain VSY017 (Supplementary Fig. S2). In other words, the deletion of *NCP1* in the yeast strain harboring two copies of *AtCPR1-CrCYB5* increased vindoline production by about 2.6-fold. Based on these results, two genomic copies of *AtCPR1* together with *NCP1* deletion contributed to the best electron transfer compatibility with CYPs of the vindoline biosynthetic pathway.

**Fig. 5:**
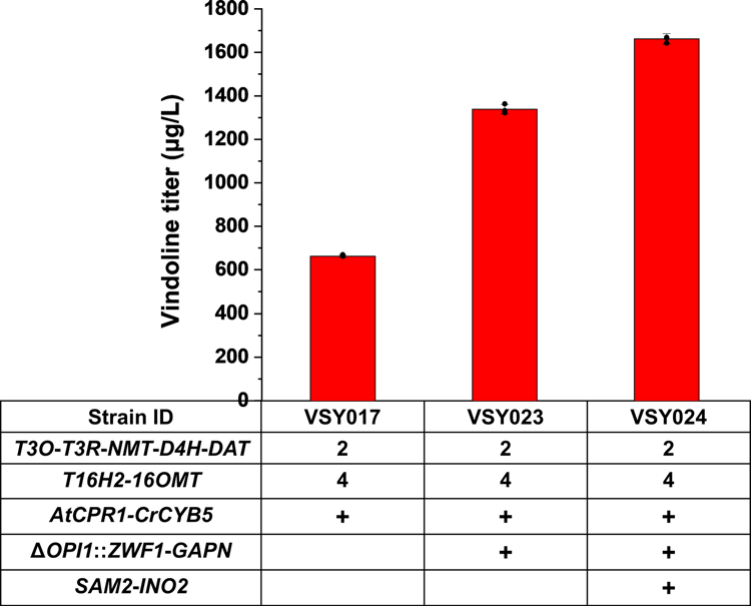
Optimization of vindoline production through ER expansion and cofactor supply enhancement (NADPH and SAM). With VSY017 as the parent strain, *OPI1* was deleted and *INO2* was overexpressed for ER expansion, *ZWF1* and *GAPN* were overexpressed to enhance NADPH supply, and *SAM2* was overexpressed to increase SAM availability. All strains were cultured in SC with 2% galactose in the presence of 50 mg/L tabersonine. Error bars represent SD of biological triplicates (*n* = 3).

As plant CYPs and CPRs are generally ER-localized membrane proteins^22^, the expansion of ER should enable higher enzymatic activities by providing more space for the folding of CYPs and CPRs^23^. Previous investigations have demonstrated that ER expansion could be achieved by the deletion of *PAH1*^23^, the deletion of *OPI1*, and the overexpression of *INO2*^24^. Although attempted multiple times, the deletion of *PAH1* was not successful in the vindoline-producing strain. The discrepancy with the previous study might result from the use of yeast strains with different genetic background. Therefore, the overexpression of *INO2* and the deletion of *OPI1* were employed in the present study. In addition, NADPH functions as the cofactor for CPRs mediated electron transfer and the increased NADPH availability has been shown to enhance CYPs activities^25^. The introduction of *GAPN*^26^ (NADP^+^-dependent glyceraldehyde-3-phosphate dehydrogenase from *Streptococcus mutans*) and overexpression of *ZWF1* were commonly employed strategies to enhance NADPH supply. Enhancing the supply of S-Adenosyl-Methionine (SAM) via the overexpression of *SAM2* has been demonstrated to improve the methyltransferase activity and accordingly the biosynthesis of secondary metabolites in yeast as well^5^. Therefore, VSY017 was further engineered by inserting the *GAPN* and *ZWF1* expression cassettes into the *OPI1* locus (VSY023), followed by the integration of *SAM2* and *INO2* expression cassettes (VSY024). The production of vindoline in VSY023 and VSY024 was increased to 1339.0 μg/L and 1662.7 μg/L, respectively (Fig. 5). Overall, the production of vindoline was increased for about fourfold (from ~417.1 μg/L in VSY015 to ~1662.7 μg/L in VSY024) via engineering of the CYP microenvironment, including CYPs-CPRs pairing, ER expansion, and NADPH supply enhancement.

### Optimization of fermentation conditions for the biosynthesis of vindoline

**Fig. 6:**
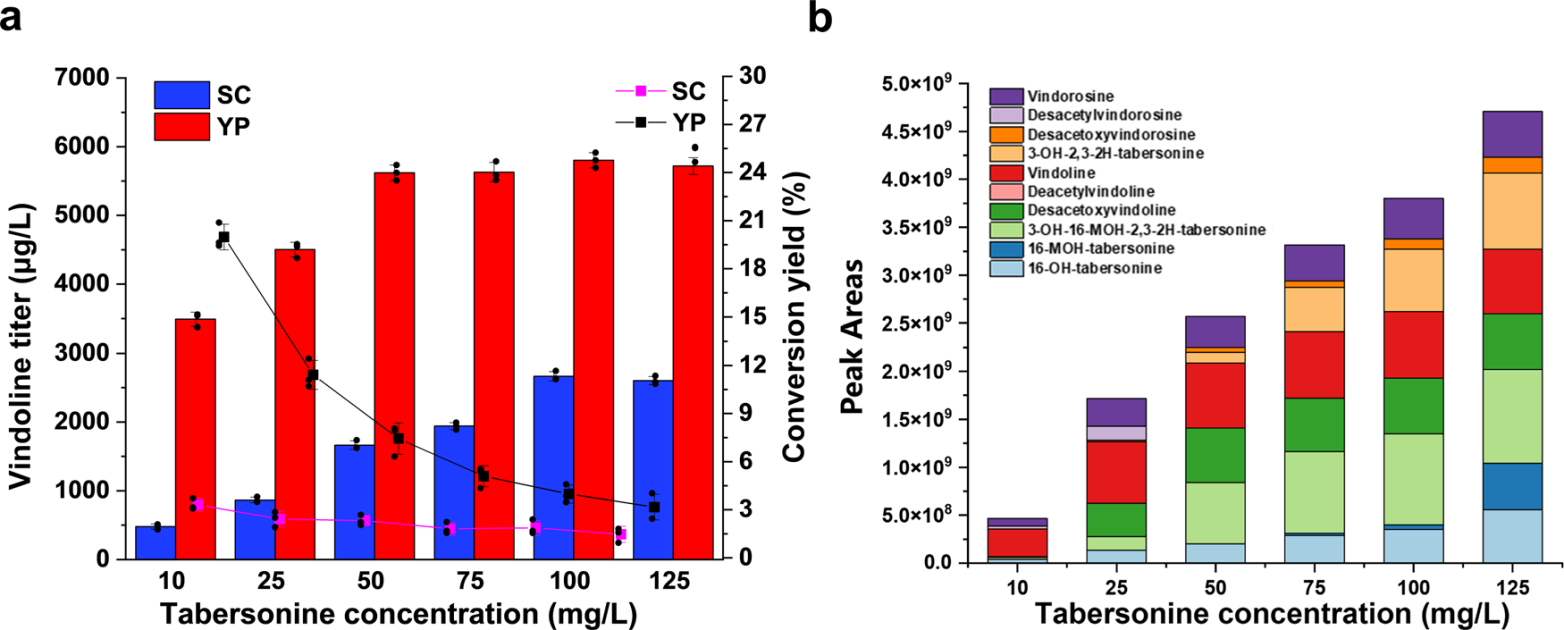
Effect of tabersonine concentration on the production of vindoline. **a** The strain (VSY024) was cultured in SC (shown in blue) or YP (shown in red) medium with 2% galactose in the presence of various concentrations of tabersonine at 30 °C. Conversion yield was calculated by the conversion of tabersonine to vindoline, with the pink line representing the conversion yield in SC medium and the black line for that in YP medium. Error bars represent SD of biological triplicates (*n* = 3). **b** The accumulation of vindoline and intermediate metabolites in strain VSY024 when different amounts of tabersonine were supplemented into YP medium. Data are average of biological triplicates (*n* = 3).

To further increase vindoline production, the fermentation conditions were briefly optimized in shaker flasks. First, SC and YP medium were investigated with different concentrations of tabersonine (10, 25, 50, 75, 100, 125 mg/L). Generally, more vindoline could be produced if a higher concentration of tabersonine was supplemented. As shown in Fig. 6a, compared with the synthetic medium, the rich medium YP led to a pronounced increase in vindoline titers (as high as ~5.8 mg/L), particularly under low tabersonine concentration. The better performance might result from improved enzyme expression levels and better cell growth in the rich medium. Nevertheless, in the YP medium, the conversion yield of tabersonine to vindoline was dramatically decreased with higher substrate supplementation (Fig. 6a).

To explore the possible reasons, the accumulation of the intermediate metabolites was analyzed when different concentrations of tabersonine were supplemented into YP medium. As shown in Fig. 6b, the percentage of 16-OH-tabersonine (light blue box) and 3-OH-16-MOH-2,3-2H-tabersonine (light green box) were increased with higher substrate supplementation, indicating that vindoline production was limited by the methyltransferases (16OMT and NMT). In addition, the percentage of 3-OH-2,3-2H-tabersonine (light orange box), an intermediate metabolite of the vindorosine pathway, was increased with higher substrate supplementation, implying that the promiscuity of T3O/T3R was another reason for low yield vindoline biosynthesis.

**Fig. 7:**
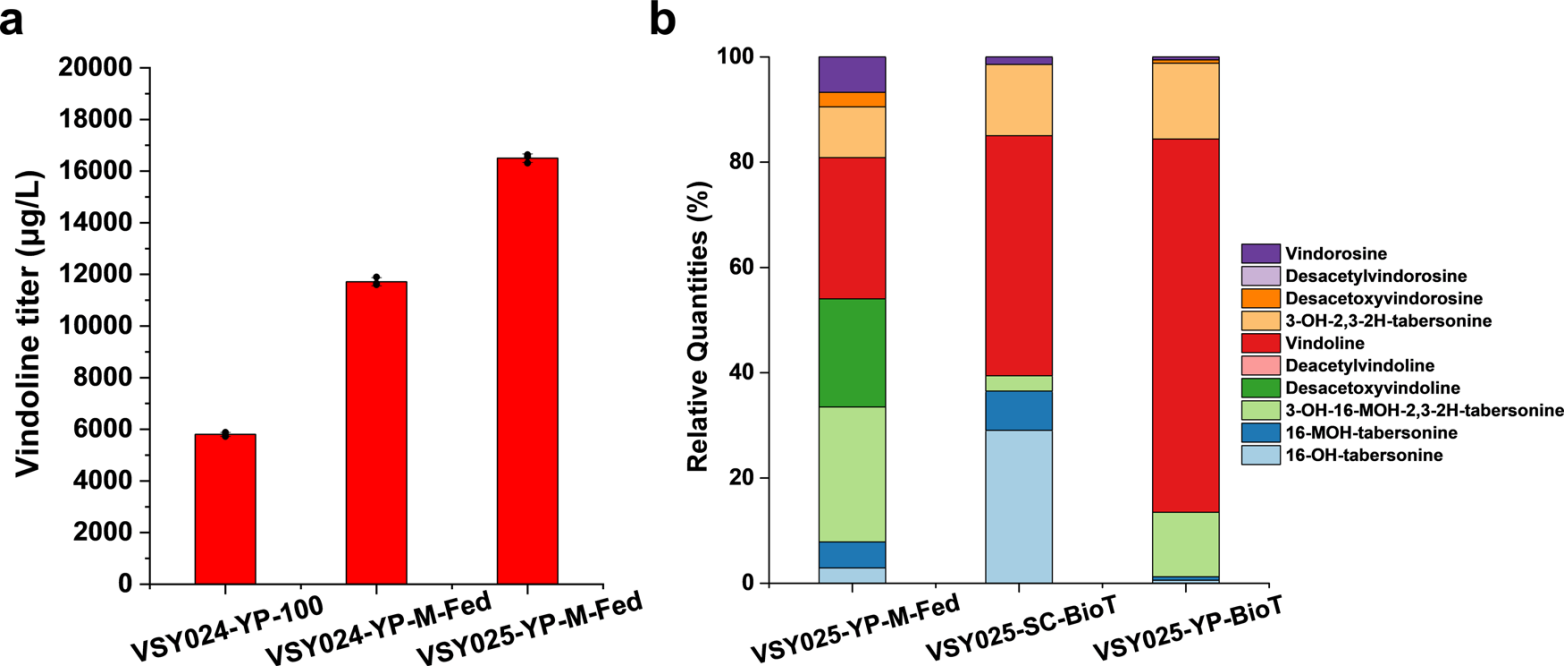
Optimization of fermentation conditions for enhanced vindoline production in yeast. **a** Enhancing vindoline production in YP medium via feeding low concentration of tabersonine (~15 mg/L) every 24 h after galactose induction. A total of ~100 mg/L tabersonine was fed into the fermentation broth. Error bars represent SD of biological triplicates. **b** The accumulation of vindoline and intermediate metabolites in strain VSY025 by intermittent feeding of low concentrations of tabersonine and high cell density yeast biotransformation (BioT). Data are average of biological triplicates (*n* = 3).

To address the dilemma between titer and yield, a low concentration of tabersonine (15 mg/L) was fed into the fermentation broth every 24 h after galactose induction in VSY024 and VSY025. With a similar amount of tabersonine supplemented (roughly 100 mg/L), the intermittent supply of the substrate at a lower concentration resulted in improved both titer and yield of vindoline, whose maximal titer reached 11.7 mg/L and 16.5 mg/L, respectively (Fig. 7a). In addition, by keeping tabersonine at a low concentration, the accumulation of vindorosine and its biosynthetic intermediates were largely decreased, while the vindoline biosynthetic intermediates (such as 3-OH-16-MOH-2,3-2H-tabersonine and desacetoxyvindoline) were still accumulated to high levels (Fig. 7b). In other words, the concerns with the promiscuity of T3O/T3R could be addressed to some extent by lowering tabersonine concentration, while the biosynthetic pathway should be further optimized to minimize intermediate accumulation.

Finally, to compare the performance of VSY025 with the previously constructed vindoline producing strain by Qu et al.^17^, which harbored the biosynthetic pathway genes on multi-copy plasmids, yeast biotransformation assay was carried out under the same conditions. With the supplementation of ~75 mg/L, while the strain constructed by Qu et al. could produce ~1.1 mg/L vindoline, VSY025 was able to produce 17.7 mg/L and 29.4 mg/L vindoline in SC medium and YP medium, respectively (Supplementary Fig. S3), indicating the advantages of the genome-integrated strains in the biosynthesis of natural products. More importantly, tabersonine biotransformation using high concentration of yeast cells not only decreased by-product formation, but also minimized intermediate accumulation, particularly in YP medium (Fig. 7b).

## Discussion

Vindoline is suggested to be one of a major alkaloid compound in *C. roseus* and has been demonstrated to possess antidiabetic activities^27^. More importantly, vindoline is condensed with catharanthine to generate the anticancer drugs vinblastine and vincristine. In the present study, the vindoline biosynthetic pathway from tabersonine was reconstituted and systematically optimized in yeast using the CRISPR/Cas9-mediated multiplex genome editing technology.

CYPs are generally considered as the bottleneck for efficient production of secondary metabolites in microbial cell factories. Vindoline production was significantly increased by improving the expression and activities of eukaryotic P450s in yeast (Fig. 4). Eukaryotic CYPs are a kind of ER located membrane proteins, thus the expansion of ER has been demonstrated to be beneficial to improve the functional expression and activities of CYPs. Based on prior studies, the knockout of *OPI1* in conjunction with the overexpression of *GAPN*, *ZWF1*, and *INO2* enhanced the vindoline titer to as high as 1662.7 μg/L in strain VSY024 (Fig. 5). Notably, the effect of *PAH1* deletion on P450 activities has been demonstrated to enhance the production of medicagenic acid and sesquiterpenoid artemisinic acid in *S. cerevisiae* CEN.PK2. Unfortunately, the knockout of *PAH1* was attempted several times without success in the yeast strain used in the present study (BY4741). The discrepancy might result from the differences in the genetic background of these two yeast strains, indicating our limited understanding of the yeast network regulating ER expansion. Considering the complexity of the ER expansion and membrane protein folding mechanisms, very limited targets (i.e. *OPI1*, *INO2*, and *PAH1*) have been identified and employed for metabolic engineering applications. Therefore, genome-scale engineering, such as RNAi-assisted genome evolution^28^ and multi-functional genome-wide CRISPR system (MAGIC)^29^, should be performed to identify novel metabolic engineering targets to enhance the functional expression of CYPs.

Although systematic metabolic engineering was carried out to improve the pathway efficiency, the vindoline conversion yield was still not satisfactory. One concern is the accumulation of intermediate metabolites. In particular, 16-OH-tabersonine and 3-OH-16-MOH-2,3-2H-tabersonine were accumulated to high levels when high concentration of tabersonine was supplemented (Fig. 6b), indicating that vindoline production was limited by the methyltransferases (16OMT and NMT). Thus, the enzymatic activities of 16OMT and NMT and/or the supply of co-factors for methyltransferases (such as SAM) should be further enhanced. Recently, the activity of methyltransferases^30^ was found to be affected by pH of the culture medium, indicating that fermentation conditions should be optimized to further increase the titer and yield of vindoline. Another concern is the formation of vindorosine as a by-product with higher substrate supplementation, which was resulted from the promiscuity of T3O for the conversion of tabersonine directly. The dilemma of titer and yield to some degree could be addressed by increasing the expression levels of *T16H2* and *16OMT* in combination of supplying low concentration of tabersonine in an intermittent manner. Nevertheless, a more promising and practical strategy is to engineer the substrate specificity of T3O. Specifically, T3O variants with enhanced activity on 16-methoxyl-tabersonine and minimized activity toward tabersonine are highly demanded for efficient and high-yield production of vindoline. For example, Sun et al. used homology modeling and molecular docking to enhance the activity and substrate specificity of CYP72A63, leading to a high-level accumulation of 11-oxo-β-amyrin and glycyrrhetinic acid in *S. cerevisiae*^31^. Due to the lack of structural information for most eukaryotic CYPs, directed evolution based on random mutagenesis and high throughput screening can greatly broaden the research and application of CYPs engineering^31,32^.

Herein, multiple metabolic engineering strategies based on CRISPR/Cas9-mediated facile genome engineering technology were employed to systematically engineer *S. cerevisiae* to produce vindoline from tabersonine. A combination of pathway optimization, CYPs-CPRs pairing, ER expansion, cofactor supply enhancement, and fermentation optimization led to the construction of a yeast cell factory to produce vindoline from tabersonine with a final titer of 16.5 mg/L, which was more than 3,800,000-fold higher than the parent strain. Therefore, the multiple metabolic engineering strategies can not only be employed to facilitate the production of other value-added secondary metabolites, but also lay a solid foundation for the establishment of de novo biosynthetic pathways for producing vindoline, and even vinblastine and vincristine.

## Methods

### Strains, media, and reagents

*Escherichia coli* Trans T1 (Transgen Biotech Co., Ltd, Beijing, China) was used for gene cloning and plasmid amplification. *E. coli* transformants were selected and cultured in LB medium containing 100 mg/L ampicillin. *S. cerevisiae* BY4741 strain was used as the host for genome engineering and vindoline production. Yeast strains were routinely cultivated in SCD (5 g/L ammonium sulfate, 1.7 g/L yeast nitrogen base without ammonium and amino acids, 0.6 g/L CSM missing the appropriate nutrients, 20 g/L glucose, and 0.2 g/L methionine) or YPD (10 g/L yeast extract, 20 g/L peptone, and 20 g/L glucose) medium. When necessary, 200 mg/L G418 sulfate (Sangon Bio-tech Co., Ltd, Shanghai, China) was supplemented. All restriction enzymes and T4 DNA ligase were purchased from NEB (Beijing, China). All chemicals were from Sigma-Aldrich (St. Louis, MO, USA) unless specifically mentioned. Tabersonine and vindoline standards were purchased from Chengdu DeSiTe Biological Technology Co., Ltd (Chengdu, China) and Yuanye Bio-tech Co., Ltd (Shanghai, China), respectively.

### Plasmid construction

Vindoline biosynthesis pathway genes including *CrT16H2*, *Cr16OMT*, *CrT3O*, *CrT3R*, *CrNMT*, *CrD4H*, *CrDAT*, *CrCYB5*, and *CrCPR* were kindly provided by Prof. Vincenzo De Luca (Brock University)^17^, *AtCPR1* from *A. thaliana*, *GlCPR* from *G. lucidum*, and *MTR2* from *Medicago* were kindly provided by Dr. Han Xiao^33^ (Shanghai Jiao Tong University) and *GuCPR1* from *G. uralensis* was provided by Prof. Chun Li^18^ (TsingHua University). The coding sequences of all the genes used in this study were listed in Supplementary Data 2 and cloned into the multiple cloning sites of the pESC series vectors (pESC-URA, pESC-LEU, pESC-LEU2d, and pESC-HIS) by digestion/ligation (MCS1: *Bam*HI/*Xho*I; MCS2: *Not*I) or Gibson Assembly. Oligonucleotides were synthesized by Tsingke Biotech Co., Ltd (Hangzhou, China). KOD-Plus-Neo DNA Polymerase (TOYOBO Biotech Co., Ltd, Tokyo, Japan) was used for gene amplification and PCR products were purified by the Gene JET PCR Purification Kit (Thermo fisher Scientific, Shanghai, China). Plasmids were extracted from *E. coli* using the AxyPrep Plasmid Miniprep Kit (Axygen, Shanghai, China) according to the manufacturer’s instructions. DNA sequencing was performed by Tsingke Biotech Co., Ltd (Hangzhou, China). All the plasmids used in this study were listed in Table 1 and the corresponding primers were provided in Supplementary Table S1.

**Table 1:** Strains and plasmids used in this study.

Plasmid/strain name	Description	Source
Plasmids		
pRS41K-SpCas9	CEN/ARS; G418; *AmpR*; *TEF1p-SpCas9-TEF1t*	^36^
p423-SpSgH	2μ; *HIS3; AmpR; SNR52p-SUP4t*	^36^
p426-SpSgH	2μ; *URA3; AmpR; SNR52p-SUP4t*	^36^
pESC-URA	2μ; *URA3; AmpR; GAL1p-*MCS1*-CYC1t; GAL10p-*MCS2*-ADH1t* (NCBI Accession AF063585)	Agilent Technologies, Inc
pESC-LEU	2μ; *LEU2; AmpR; GAL1p*-MCS1-*CYC1t; GAL10p*-MCS2-*ADH1t* (NCBI Accession AF063849)	Agilent Technologies, Inc
pESC-LEU2d	2μ; *LEU2d; AmpR; GAL1p*-MCS1-*CYC1t; GAL10p*-MCS2-*ADH1t* (Addgene plasmid #20120)	^9^
pESC-HIS	2μ; *HIS3; AmpR; GAL1p*-MCS1-*CYC1t; GAL10p*-MCS2-*ADH1t* (NCBI Accession AF063850)	Agilent Technologies, Inc
pESC-LEU2d-*T16H2*-*16OMT*	2μ; *LEU2d; AmpR; GAL1p*-*T16H2-CYC1t; GAL10p*-*16OMT*-*ADH1t*	This study
pESC-URA-*CrCPR*-CrCYB5	2μ; *URA3; AmpR; GAL1p-CrCPR*-*CYC1t; GAL10p*-*CrCYB5*-*ADH1t*	This study
pESC-HIS-*NMT*-*T3R*	2μ; *HIS3; AmpR; GAL1p-NMT*-*CYC1t; GAL10p*-*T3R-ADH1t*	This study
pESC-URA-*T3O*	2μ; *URA3; AmpR; GAL1p*-*T3O-CYC1*	This study
pESC-URA-*D4H*-*DAT*	2μ; *URA3; AmpR; GAL1p*-*D4H*-*CYC1t; GAL10p*-*DAT*-*ADH1t*	This study
pESC-URA-*AtCPR1*-*CrCYB5*	2μ; *URA3; AmpR; GAL1p*-*AtCPR1*-*CYC1t; GAL10p*-*CrCYB5*-*ADH1t*	This study
pESC-URA-*GuCPR1*-*CrCYB5*	2μ; *URA3; AmpR; GAL1p*-*GuCPR1*-*CYC1t; GAL10p*-*CrCYB5*-*ADH1t*	This study
pESC-URA-*GlCPR*-*CrCYB5*	2μ; *URA3; AmpR; GAL1p*-*GLCPR*-*CYC1t; GAL10p*-*CrCYB5*-*ADH1t*	This study
pESC-URA-*MTR2*-*CrCYB5*	2μ; *URA3; AmpR; GAL1p-MTR2*-*CYC1t; GAL10p*-*CrCYB5*-*ADH1t*	This study
pESC-URA-*SAM*2-*INO2*	2μ; *URA3; AmpR; GAL1p*-*SAM2*-*CYC1t; GAL10p*-*INO2*-*ADH1t*	This study
pRS423-*ZWF1*-*GAPN*	2μ; *HIS3; AmpR; PGK1p*-*ZWF1*-*CYC1t; TEF1p*-*GAPN*-*ADH1t*	This study
Strains		
BY4741	MATa: *his3∆1 leu2∆0 met15∆0 ura3∆0*	ATCC
VSY001	BY4741 with pRS41K-SpCas9	This study
VSY002	VSY001*-GAL1p*-*CrCPR*-*CYC1t-GAL10p*-*CrCYB5*-*ADH1t*	This study
VSY003	VSY002-*GAL1p-T16H2*-*CYC1t-GAL10p*-*16OMT*-*ADH1t*	This study
VSY004	VSY003-*GAL1p*-*NMT*-*CYC1t-GAL10p*-*T3R*-*ADH1t*	This study
VSY005	VSY004-*GAL1p*-*T3O*-*CYC1t*	This study
VSY006	VSY005-*GAL1p*-*D4H*-*CYC1t-GAL10p*-*DAT*-*ADH1t*	This study
VSY007	VSY006/pESC-LEU2d-*T16H2*-*16OMT*	This study
VSY008	VSY006-*GAL1p*-*T16H2*-*CYC1t-GAL10p*-*16OMT*-*ADH1t*	This study
VSY009	VSY008-*GAL1p*-*T3O*-*CYC1t-GAL10p*-*T3R*-*ADH1t*- *GAL1p*-*NMT*-*CYC1t- GAL1p*-*D4H*-*CYC1t-GAL10p*-*DAT*-*ADH1t*	This study
VSY014	VSY009*-GAL1p*-*T16H2*-*CYC1t-GAL10p*-*16OMT*-*ADH1t*	This study
VSY015	VSY014-*GAL1p-T16H2*-*CYC1t-GAL10p*-*16OMT*-*ADH1t*	This study
VSY016	VSY015-*GAL1p*-*T16H2*-*CYC1t-GAL10p*-*16OMT*-*ADH1t*	This study
VSY017	VSY015-Δ*CrCPR*::*AtCPR1*	This study
VSY018	VSY015-Δ*CrCPR*::*GuCPR1*	This study
VSY019	VSY015-Δ*CrCPR*::*GlCPR*	This study
VSY020	VSY015-Δ*CrCPR*::*MTR2*	This study
VSY021	VSY015*-GAL1p*-*CrCPR*-*CYC1t-GAL10p*-*CrCYB5*-*ADH1t*	This study
VSY022	VSY015-*GAL1p*-*AtCPR1*-*CYC1t-GAL10p*-*CrCYB5*-*ADH1t*	This study
VSY023	VSY017-Δ*OPI*:: *PGK1p*-*ZWF1*-*CYC1t-TEF1p*-*GAPN*-*ADH1t*	This study
VSY024	VSY023-*GAL1p*-*SAM2*-*CYC1t*-*GAL10p*-*INO2*-*ADH1t*	This study
VSY017-2	VSY017- *GAL1p*-*AtCPR1*-*CYC1t-GAL10p*-*CrCYB5*-*ADH1t*	This study
VSY017-3	VSY017-Δ*NCP1*::*GAL1p*-*AtCPR1*-*CYC1t-GAL10p*-*CrCYB5*-*ADH1t*	This study
VSY017-4	VSY017-Δ*NCP1*::*GAL1p*-*GuCPR1*-*CYC1t-GAL10p*-*CrCYB5*-*ADH1t*	This study
VSY017-5	VSY017-Δ*NCP1*::*GAL1p*-*GlCPR*-*CYC1t-GAL10p*-*CrCYB5*-*ADH1t*	This study
VSY017-6	VSY017-Δ*NCP1*::*GAL1p*-*MTR2*-*CYC1t-GAL10p*-*CrCYB5*-*ADH1t*	This study
VSY017-7	VSY017-Δ*NCP1*::*GAL1p*-*CrCPR*-*CYC1t-GAL10p*-*CrCYB5*-*ADH1t*	This study
VSY025	VSY024-Δ*NCP1*::*GAL1p*-*AtCPR1*-*CYC1t-GAL10p*-*CrCYB5*-*ADH1t*	This study

### Strain construction

All genetic modifications in yeast were carried out via the CRISPR/Cas9-mediated genome editing method^34,35^ and the schematic overview was briefly demonstrated in Supplementary Fig. S1. Genome integration loci and the corresponding gRNA plasmids (Supplementary Table S2) were constructed in our previous studies^29,36–38^. For the construction of strains VSY001-VSY024, target gene expression cassettes were amplified by PCR with 40 bp homology arms to the target chromosomal loci and co-transformed with the corresponding gRNA plasmids to the Cas9 expressing yeast strains using the LiAc/SS carrier DNA/PEG method^39^. All the strains constructed in this study were listed in Table1.

### Fermentation conditions

For vindoline and other intermediates quantification, a single colony was picked from YPD or SCD agar plates and subcultured at 30 °C and 250 rpm for 24 h. Then, 100 μL seed culture was inoculated into a 250 mL flask containing 10 mL fresh YPD or SCD medium. After culturing at 30 °C for 24 h, the yeast cells were harvested and resuspended in SC or YP medium supplemented with 2 % galactose (SCG or YPG) and 10–150 mg/L tabersonine. The induced yeast cells were cultivated in a 250-mL shake flask with 10 mL volume at 30 °C for additional 168 h. Intermittent feeding of tabersonine was performed for the best performing *S. cerevisiae* strain VSY024, following a similar protocol, except that 15 mg/L tabersonine was supplemented every 24 h after induction by galactose. All the experiments were performed in biological triplicates.

### Yeast biotransformation assays

A single colony of VSY025 was inoculated in 2 mL SCD or YPD medium and cultured at 30 °C overnight. Yeast cells were harvested and inoculated into fresh 2 mL SCG or YPG medium and induced at 30 °C for 24 h. The induced yeast cells were harvested and suspended in 1 mL biotransformation buffer (10 mM Tris-HCl at pH 7.5 and 1 mM EDTA) containing 20 μM tabersonine and incubated at 30 °C for 12 h. After the biotransformation assays, the production of vindoline and other intermediates were quantified by LC–MS.

### Analytical methods

The culture broth was centrifuged at 11,000 × *g* for 10 min. For the strains with low vindoline production (VSY006-VSY009), 20 mL supernatant was freeze-dried and then reconstituted in 1 mL water. The resultant mixture was extracted by 1 mL ethyl acetate twice. After being dried by a rotary evaporator, the samples were resuspended in 150 μL methanol and passed through a 0.22 µm membrane filter for LC–MS analysis. For the high-producing strains (VSY010-VSY024), 400 μL supernatant was extracted by 1 mL ethyl acetate twice, and the organic phase was passed through a 0.22 µm membrane filter and subject to LC–MS analysis.

HPLC-MS (SHIMADZU LC-MS/MS 8045, Tokyo, Japan) analysis was used for the separation, identification, and quantification of vindoline and other intermediates through a HyPURITY^TM^ C18 HPLC (150 mm × 4.6 mm, 3 μm, Thermo Scientific) column with an ESI ion source equipped with a triple quadrupole mass analyzer. Vindoline and tabersonine were monitored using LC–MS multiple-reaction monitoring (MRM) with the following parameters: vindoline with a collision energy of 28 eV and an *m/z* transition from 457.05 to 188.05, tabersonine with a collision energy of 22 eV and an *m*/*z* transition from 337.40 to 305.05, other intermediates with a positive scanned mode from *m/z* 50 to 800. The desolvation line temperature was held at 200 °C, with a spray voltage of 1.8 kV and an atomizing gas flow rate of 3 L/min. The mobile phase was consisted of 0.1% formic acid solution (solvent A) and methanol (solvent B). The following gradient elution program was used: 90% to 10% solvent A over 20 min and returned to 90% solvent A over another 20 min with a constant flow rate of 0.2 mL/min.

### Statistics and reproducibility

General data analysis (means and standard deviation) was performed primarily by OriginPro 2021 V.9.8.0.200. All experiments were performed with biological triplicates and values were expressed as means ± standard errors.

### Reporting summary

Further information on research design is available in the Nature Research Reporting Summary linked to this article.

## Supplementary information

Supplementary Information

Descriptions of Additional Supplementary Files

Supplementary Data 1

Supplementary Data 2

Reporting Summary

## Data Availability

All data are included in the manuscript, Supplementary Material, Supplementary Data 1, and Supplementary Data 2. Any other data are available from the corresponding author upon reasonable requests.
