# Supplementary material for: Efficient production of vindoline from tabersonine by metabolically engineered Saccharomyces cerevisiae
Source: Commun Biol. 2021 Sep 16;4:1089. doi: 10.1038/s42003-021-02617-w (PMC8446080; doi:10.1038/s42003-021-02617-w)
Supplement: Supplementary file 1 — Supplementary Information [file 42003_2021_2617_MOESM1_ESM.pdf]

Supplementary Material

Efficient Production of Vindoline from Tabersonine by Metabolically Engineered *Saccharomyces cerevisiae*

Tengfei Liu[1,2], Ying Huang[3], Lihong Jiang[1], Chang Dong[1,2], Yuanwei Gou[1], Jiazhang Lian [1,2*]

[1] Key Laboratory of Biomass Chemical Engineering of Ministry of Education, College of Chemical and Biological Engineering, Zhejiang University, Hangzhou, 310027, China

[2] Hangzhou Global Scientific and Technological Innovation Center, Zhejiang University, Hangzhou, 310027, China

[3] School of Chinese Materia Medica, Beijing University of Chinese Medicine, Beijing, 100102, China

***Correspondence**

Prof. Jiazhang Lian

Email: jzlian@zju.edu.cn

Key Laboratory of Biomass Chemical Engineering of Ministry of Education, College of Chemical and Biological Engineering, Zhejiang University, Hangzhou, 310027, China

Supplementary Figures

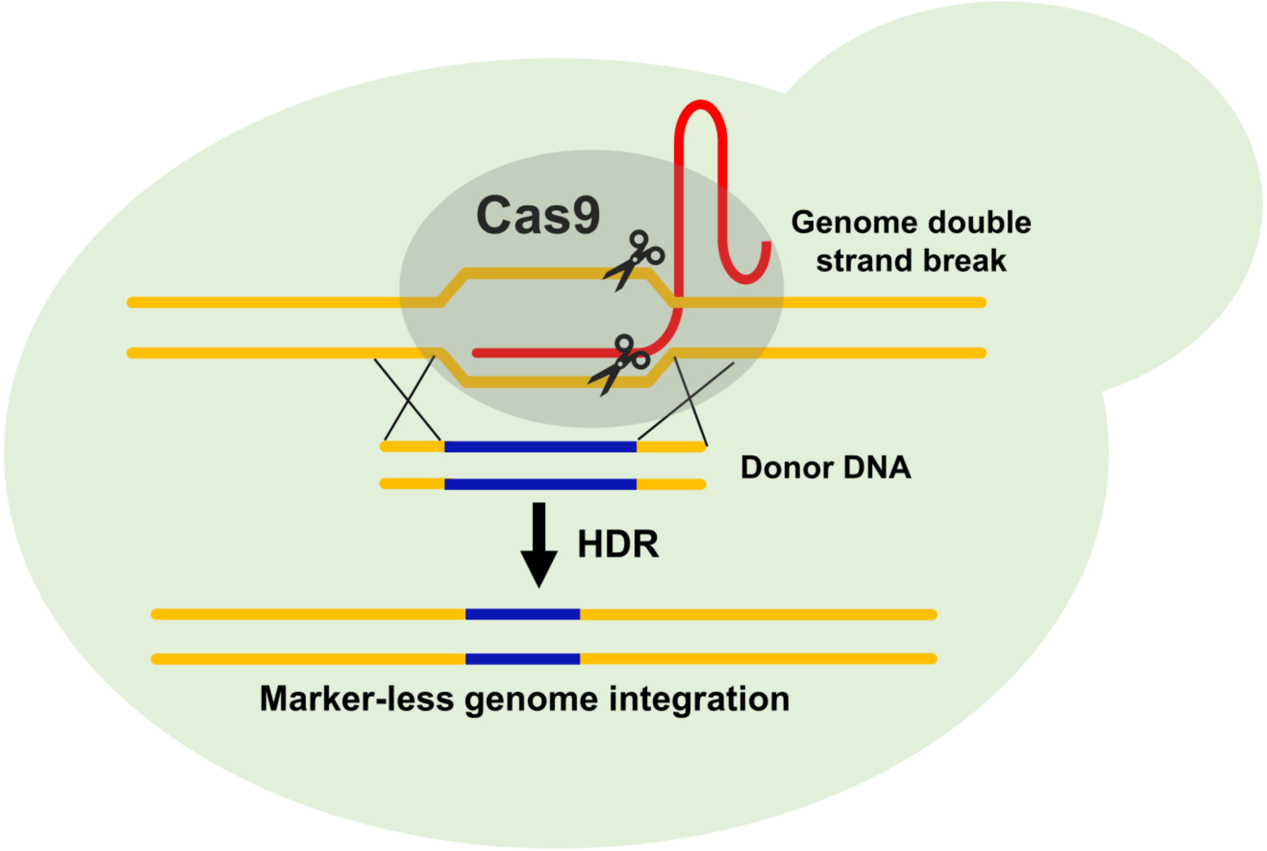

Supplementary Fig. S1 A schematic overview of CRISPR/Cas9-mediated marker-less genome integration for the construction of the vindoline-producing yeast cell factories. The Cas9 nuclease was guided to a specific genomic locus by a sgRNA (shown in red) and led to the formation of a double strand break. The resultant genome double strand break was repaired via HDR. The donor DNA contained the heterologous gene expression cassettes (shown in blue) with 40 bp upstream and downstream homologous arms (shown in yellow) were integrated into the genome of *S. cerevisiae*. HDR: homology directed repair.

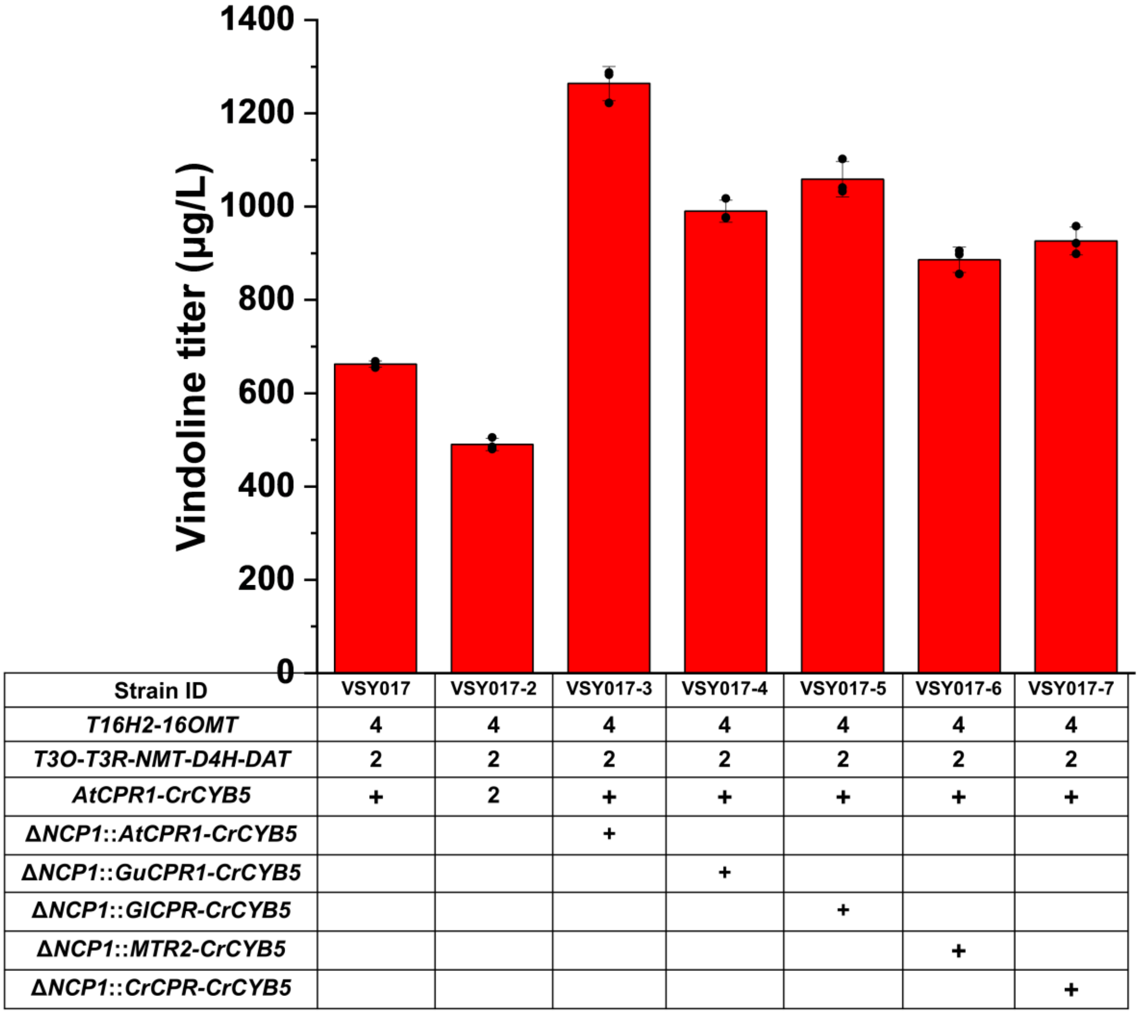

Strain ID	VSY017	VSY017-2	VSY017-3	VSY017-4	VSY017-5	VSY017-6	VSY017-7
T16H2-16OMT	4	4	4	4	4	4	4
T3O-T3R-NMT-D4H-DAT	2	2	2	2	2	2	2
AtCPR1-CrCYB5	+	2	+	+	+	+	+
Δ*NCP1*::*AtCPR1-CrCYB5*			+				
Δ*NCP1*::*GuCPR1-CrCYB5*				+			
Δ*NCP1*::*GlCPR-CrCYB5*					+		
Δ*NCP1*::*MTR2-CrCYB5*						+	
Δ*NCP1*::*CrCPR-CrCYB5*							+

Supplementary Fig. S2 Characterization of various CPRs for high vindoline production in strain VSY017. Five CPRs with different origins (*CrCPR* from *C. roseus*, *AtCPR1* from *A. thaliana*, *GuCPR1* from *G. uralensis*, *GlCPR* from *G. lucidum*, and *MTR2* from *Medicago*) were integrated into the *NCP1* locus of the engineered yeasts and their effects on vindoline production were investigated. All strains were cultured in SC medium with 2% galactose in the presence of 50 mg/L tabersonine. Error bars represent SD of biological triplicates (n=3).

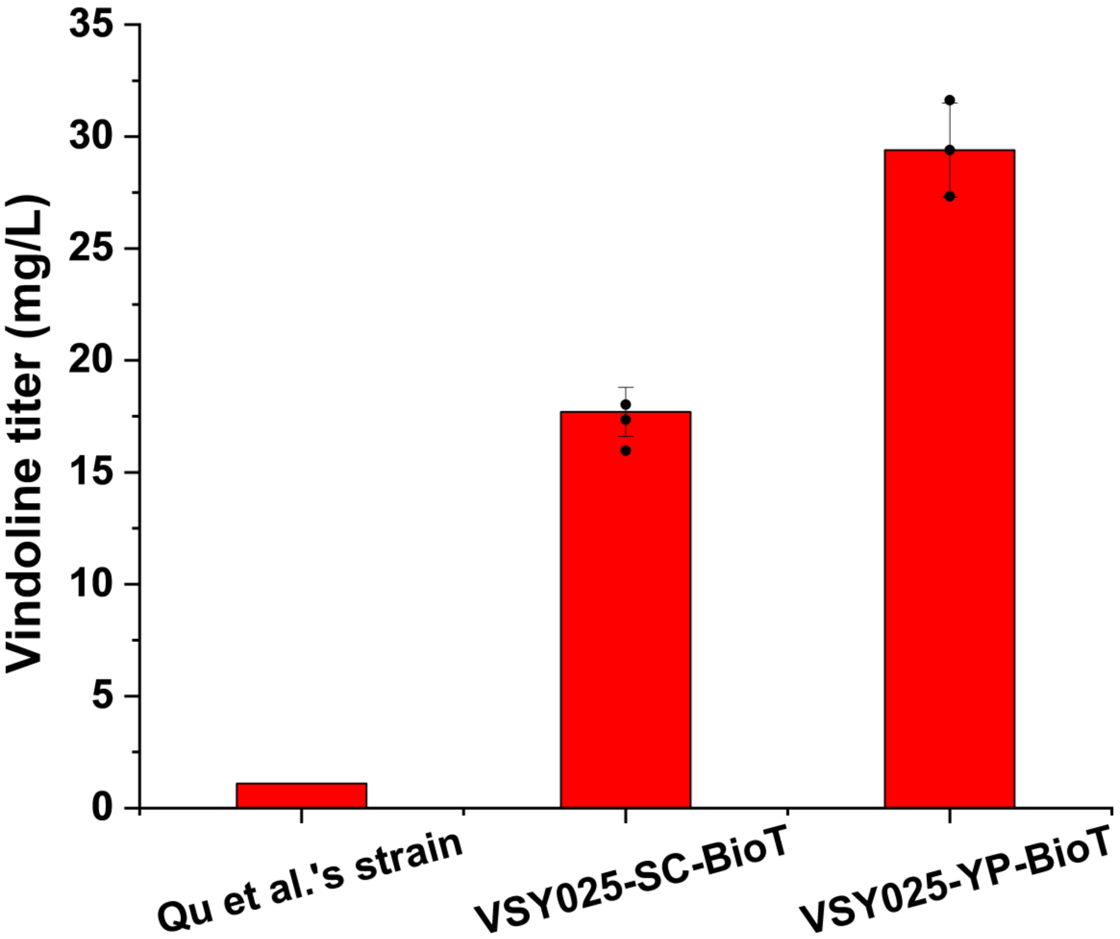

Supplementary Fig. S3 The production of vindoline in strain VSY025 using high cell density yeast biotransformation (BioT). To demonstrate the advantages of the genome integrated strains in producing natural products, the production of vindoline was compared with the previously constructed strain, which included the same set of biosynthetic pathway genes on multi-copy plasmids (Qu et al.'s strain), under the same biotransformation conditions. The samples of the biotransformation assays were taken in 12 h. Error bars represent SD of biological triplicates (n=3).

Supplementary Tables

Supplementary Table S1 List of primers used in this study

Primer name	Sequence (5′-3′)
**For plasmids construction**	
T16H2-BamHI-F	aaccccggatccatggagttgtattatttttcc
T16H2-XhoI-R	gcttactcgagctaatatttacctttgagagaa
16OMT-NotI-F	atccttgtaatccatcgatactagttcaaggataaacctcaatgaga
16OMT-NotI-R	attcaaccctcactaaagggcggccgcatggatgttcaatctgaggagttc
T3R-BamHI-F	aaccccggatccatggctgcaaagtcagtgaag
T3R-XhoI-R	gcttactcgagttagggtgatttgaaagtgttt
NMT-NotI-F	ccttgtaatccatcgatactagttattgattttcgtcccgtaactacag
NMT-NotI-R	cgaattcaaccctcactaaagggcggccgcatggaagagaagcaggagaaggt
T3O-BamHI-F	aaccccggatccatggagtttcatgaatcttct
T3O-XhoI-R	gcttactcgagcatgcataggacgtagcgatta
D4H-BamHI-F	aaccccggatccatgaaggacttgaactttcatg
D4H-XhoI-R	gcttactcgagattgtttaacctgaaaggagat
DAT-NotI-F	tccttgtaatccatcgatactagtatgaaggacttgaactttcatg
DAT-NotI-R	attcgaattcaaccctcactaaagggcggccgcattgtttaacctgaaaggaga
CrCPR-BamHI-F	taacgtcaaggagaaaaaaccccggatccatggattctagctcggagaag
CrCPR-XhoI-R	tagctagccgcggtaccaagcttactcgagtcaccagacatctcggagata
CrCYB5-NotI-F	ttgtaatccatcgatactagtttacttctccttagtatagtg
CrCYB5-NotI-R	cgaattcaaccctcactaaaggatggcgtcggatcagaaatt
AtCPR1-BamHI-F	taacgtcaaggagaaaaaaccccggatccatgacttctgctttgtatgct
AtCPR1-XhoI-R	agctagccgcggtaccaagcttactcgagtcaccagacatctctgaggta
GuCPR1-BamHI-F	taacgtcaaggagaaaaaaccccggatccatgacttcgaattccgatttg
GuCPR1-XhoI-R	gctagccgcggtaccaagcttactcgagtcaccagacatccctgaggtaa
GlCPR-BamHI-F	taacgtcaaggagaaaaaaccccggatccatggcgtccaccgacaccgtc
GlCPR-XhoI-R	agctagccgcggtaccaagcttactcgagtcagctccaaacatccgtcaa
MTR2-BamHI-F	taacgtcaaggagaaaaaaccccggatccatgcaagattcaagctcaatg
MTR2-XhoI-R	agctagccgcggtaccaagcttactcgagttaccatacatcacgcaaata
SAM2-BamHI-F	aacgtcaaggagaaaaaaccccggatccatgtccaagagcaaaactttct
SAM2-XhoI-R	agctagccgcggtaccaagcttactcgagttaaaattccaatttctttggt
INO2-NotI-F	catccttgtaatccatcgatactagttcaggaatcatccagtatgtgct
INO2-NotI-R	tcgaattcaaccctcactaaagggcggccgcatgcaacaagcaactgggaacg
ZWF1-BamHI-F	aaccccggatccatgagtgaaggccccgtcaaa
ZWF1-XhoI-R	gcttactcgagctaattatccttcgtatctt

GAPN-NotI-F	gctcagatcagatcttgttttactagtgtcactttatgtcaaagacaa
GAPN-NotI-R	tctaatctaagttttaattacaagcggccgcatgacaaaacaatacaaaaact
**For genome integration**	
INT1-*CrCPR-CrCYB5*-F	gaaagaaaaactaacacattaatgtagttttaaaatttcagagcgacctcatgctatac
INT1-*CrCPR-CrCYB5*-R	taatttttattctagcatatatttaagtttgtttgcgaaacttcgagcgtcccaaaacc
INT2-*T16H2-16OMT*-F	agcaataaacgtgaaatctgttttgtatatatatttgcaggagcgacctcatgctatac
INT2-*T16H2-16OMT*-R	ggttttgggacgctcgaagcggataatttgagcaatgatagtatgaaatgccagattac
INT3-*D4H-DAT*-F	gggctcaagaagacgttgataataagaacacggaaaatgggagcgacctcatgctatac
INT3-*D4H-DAT*-R	tggttggtattgttaatgttactattactgttcttgtcctcttcgagcgtcccaaaacc
INT4-*T3R-NMT* -F	ttttaggatattgacgccaagcgtgcgtctgatttctacgttcaggctgcgcaactgtt
INT4-*T3R-NMT* -R	gtttaataatgatctgtattgctggctcaatccacgtaagagtgagctgataccgctcg
INT5-*T3O* -R	accggtaccggaggagaccgctataaccggtttgaatttagagcgacctcatgctatac
INT5-*T3O* -R	ttcgaatgatgaacttgcttgctgtcaaacttctgagttgcttcgagcgtcccaaaacc
INT6(VSY008,009)-*T16H2-16OMT*-F	tattctactaaaaacacatcagtagtcacagaagtcacagttcaggctgcgcaactgtt
INT6(VSY008,009)-*T16H2-16OMT*-R	attttttcattaatacacctgtgttagttatgattgccagagtgagctgataccgctcg
INT7(VSY009,014-016)-*T3R-NMT*-F	gagagaaattaaacttggttggggttaattatttgatgggttcaggctgcgcaactgtt
INT7(VSY009,014-016)-*T3R-NMT*-R	tattgataaaggttttgtagaatatttattatcgatacctagtgagctgataccgctcg
INT8(VSY009,014-016)-*T3O* -F	acactcgcgagaaccaaaacaaggatcccctaaacccagtttcaggctgcgcaactgtt
INT8(VSY009,014-016)-*T3O*-R	ccgcccccttttccccagatcgctgagataggtgcctcacagtgagctgataccgctcg
INT9(VSY009,014-016)-*D4H-DAT*-F	aggcaatgagcgaaagcgactgaagccgagagatgtgcccttcaggctgcgcaactgtt
INT9(VSY009,014-016)-*D4H-DAT*-R	gactagtatcatccgtcaagaagaacaagaacaagaacaaagtgagctgataccgctcg
INT10(VSY014)-*T16H2-16OMT*-F	gttttcttatttctttctttttaaaaaactttcttaatatttcaggctgcgcaactgtt
INT10(VSY014)-*T16H2-16OMT*-R	ttgagaaaaaaagtgtatatcattacattactttacaccaagtgagctgataccgctcg
INT11(VSY015)-*T16H2-16OMT*-F	tatgtattttgcagcttctactttctgcaatgtgatgccgttcaggctgcgcaactgtt
INT11(VSY015)-*T16H2-16OMT*-R	cagtaactaatcgcaaacaaatcaggcatctgtgtatattagtgagctgataccgctcg
INT12(VSY016)-*T16H2-16OMT*-F	ttacgtgtcatttattatgggttcagaaattatgtgttaattcaggctgcgcaactgtt
INT12(VSY016)-*T16H2-16OMT*-R	acaattttggtggcgttgaaattgatgccggaatttgcccagtgagctgataccgctcg
Δ*CrCPR*(VSY017-020)-*CPRs*-F	gaaagaaaaactaacacattaatgtagttttaaaatttcagagcgacctcatgctatac
Δ*CrCPR*(VSY017-020)-*CPRs*-R	taatttttattctagcatatatttaagtttgtttgcgaaacttcgagcgtcccaaaacc
INT13(VSY021)-*CrCPR-CrCYB5* -F	ctatgtgacgctgtgtattctttgttgtagttatgctccattcaggctgcgcaactgtt
INT13(VSY021)-*CrCPR*-R	tgtacgctatacatttacgtgctgagctcctaggaaagctagtgagctgataccgctcg
INT13(VSY022)-*AtCPR1-CrCYB5*-F	ctatgtgacgctgtgtattctttgttgtagttatgctccattcaggctgcgcaactgtt
INT13(VSY022)-*AtCPR1-CrCYB5*-F	tgtacgctatacatttacgtgctgagctcctaggaaagctagtgagctgataccgctcg
Δ*OPI1*(VSY023)::*ZWF1-GAPN*-F	attcaaaggtaaagagggtccgataataatggtagttcaattcaggctgcgcaactgtt
Δ*OPI1*(VSY023)::*ZWF1-GAPN*-R	agcgatctgcacttagccaagaaagcatatcaggccagaaagtgagctgataccgctcg
INT14(VSY024)-*SAM2-INO2*-F	ctaccaaggttgttgagggaacactggggcaataggctgtgagcgacctcatgctatac
INT14(VSY024)-*SAM2-INO2*-R	ctgttacttcttgcagacatcagacatactattgtaattccttcgagcgtcccaaaacc
Δ*NCP1*(VSY017-1)-*CPRs*-F	gatctactgtcgcacatatcatgccgtttggaatagacaactatctcagcgatctgtct

Δ*NCP1*(VSY017-1)-*CPRs*-R	ttggtatctacctgaagtcttgagcatcttgattagctctttcaacatttccgtgtcgc
INT15(VSY017-2)-*AtCPR1*-*CYB5*-F	ttacgtgtcatttattatgggttcagaaattatgtgttaattcaggctgcgcaactgtt
INT15(VSY017-2)-*AtCPR1*-*CYB5*-R	acaattttggtggcgttgaaattgatgccggaatttgcccagtgagctgataccgctcg
Δ*NCP1*(VSY017-1, 3-7; VSY025)-*CPRs*-F	tgtagcttaagtttgcgttcctgtagtggtcaccgctacggatgtgctgcaaggcgatt
Δ*NCP1*(VSY017-1, 3-7; VSY025)-*CPRs*-R	ctggatcttcgagcgtcccgtggttgaacaaccgcgccgaaggcttgatcagtgggctg

* Restriction sites are underlined.

Supplementary Table S2 List of sgRNAs used in this study

SgRNAs	Sequences (5′-3′)	Chromosomal loci
Sg-INT1	AATCCGAACAACAGAGCATA	ChrXVI: 776,883-776,902
Sg-INT2	GCGCCACAGTTTCAAGGGTC	ChrXIV:28,250-28,269
Sg-INT3	GGTTTTCATACTGGGGCCGC	ChrVI: 237,073-237,092
Sg-INT4	CGATACAGTGGCTGCTCATG	ChrXI: 93,963-93,982
Sg-INT5	TTGTCACAGTGTCACATCAG	ChrXII: 839,660-839,679
Sg-INT6	AAGTCACGTATATAAGCTAG	ChrVI: 210,532-210,551
Sg-INT7-	TATATTAATTTGCAACCGCA	ChrXII: 498,543-498,562
Sg-INT8	ACCCGCATACGGTTCTCCCG	ChrXVI: 641,486-641,505
Sg-INT9	TATATTAATTTGCAACCGCA	ChrXIV: 662,242-662,261
Sg-INT10	ACACGTTTGTGGTTATAAGG	ChrIV: 561,137-561,156
Sg-INT11	GTAAAACACCTATAGCACTG	ChrVII: 320,896-320,915
Sg-INT12	GTTGAAATATAAGTAACCCT	ChrX: 415,611-415,630
Sg-Δ*CrCPR*::CPRs	CCTGGAGGACGAATTCACAA	*CrCPR*:1,555-1,574
Sg-INT13	TGGCATTGAGATTCCAACG	ChrXV: 544,591-544,610
Sg-Δ*OPI1*	TGTCGCGGGCGATTGCCAAG	ChrVIII: 67,100-67,119
Sg-INT14	CGCCATTCAAGAGCAGCAAC	ChrX: 236,843-236,862
Sg-INT15	GTTGAAATATAAGTAACCC	ChrX: 415,611-415,630
Sg-Δ*NCP1*	CGGACAAGTCAAATTCAGAG	ChrVIII 190,543-192,618
