# Supplementary material for: Efficient production of vindoline from tabersonine by metabolically engineered Saccharomyces cerevisiae
Source: Commun Biol. 2021 Sep 16;4:1089. doi: 10.1038/s42003-021-02617-w (PMC8446080; doi:10.1038/s42003-021-02617-w)
Supplement: Supplementary file 2 — Descriptions of Additional Supplementary Files [file 42003_2021_2617_MOESM2_ESM.pdf]

Description of Additional Supplementary Files

File name: Supplementary Data 1.
Description: Source data underlying graphs and charts.

File name: Supplementary Data 2.
Description: DNA sequences of genes used in this study.
